# Supplementary material for: Content-rich biological network constructed by mining PubMed abstracts
Source: BMC Bioinformatics. 2004 Oct 8;5:147. doi: 10.1186/1471-2105-5-147 (PMC528731; doi:10.1186/1471-2105-5-147)
Supplement: Additional File 2 — The original results of the above study (non-essential files are deleted to keep the file size under the limit set by BMC bioinformatics). [file 1471-2105-5-147-S2.bz2 › chilibotAdditionalFile2/dip05/175ID7592797E594/html/IFNAR1_IFNA2.html]

 


 **IFNAR1** and **IFNA2** 
  
Found 467 abstracts in PubMed, retrieved 05.  
 

 What does Google say? 
 PDF only 
| .edu only 

---

- Clin Exp Immunol, 2003   **Anti cytokine autoantibodies in autoimmunity preponderance of neutralizing autoantibodies against interferon alpha **[IFNAR1]**, interferon omega and interleukin 12 in patients with thymoma and or myasthenia gravis.** .
  We have screened for spontaneous anticytokine autoantibodies in patients with infections, neoplasms and autoimmune diseases, because of their increasingly reported co occurrence.
  We tested for both binding and neutralizing autoantibodies to a range of human cytokines, including interleukin 1alpha IL 1alpha , IL 1beta, IL 2, IL 4, IL 6, IL 8, IL 10, IL 12, IL 18, interferon alpha2 **[IFNA2]** IFN alpha2 , IFN omega, IFN beta, IFN gamma, tumour necrosis factor alpha TNF alpha , transforming growth factor beta 1 TGF beta1 and granulocyte macrophage colony stimulating factor GM CSF , in plasmas or sera.
  With two notable exceptions described below, we found only occasional, mostly low titre, non neutralizing antibodies, mainly to GM CSF.
  also to IL 10 in pemphigoid.
  Strikingly high titre, mainly IgG, autoantibodies to IFN alpha2, IFN omega and IL 12 were common at diagnosis in patients with late onset myasthenia gravis LOMG , thymoma T but no MG TMG and especially with both thymoma and MG together TMG .
  The antibodies recognized other closely related type I IFN alpha subtypes, but rarely the distantly related type I IFN beta, and never detectably the unrelated type II IFN gamma.
  Antibodies to IL 12 showed a similar distribution to those against IFN alpha2, although prevalences were slightly lower.
  correlations between individual titres against each were so modest that they appear to be entirely different specificities.
  Neither showed any obvious correlations with clinical parameters including thymoma histology and HLA type, but they did increase sharply if the tumours recurred.
  These antibodies neutralized their respective cytokine in bioassays in vitro.
  although they persisted for years severe infections were surprisingly uncommon, despite the immunosuppressive therapy also used in most cases.
  These findings must hold valuable clues to autoimmunizing mechanisms in paraneoplastic autoimmunity.
